# Causes of peroneal neuropathy associated with orthopaedic leg lengthening in different canine models

**DOI:** 10.1007/s11751-018-0313-2

**Published:** 2018-05-25

**Authors:** Natalia A. Shchudlo, Tatyana N. Varsegova, Mikhail M. Shchudlo, Mikhail A. Stepanov, Andrey A. Yemanov

**Affiliations:** Russian Ilizarov Scientific Center for Restorative Traumatology and Orthopaedics, 6, M.Ulyanova Street, Kurgan, Russian Federation 6640014

**Keywords:** Peroneal nerve, Neuropathy, Leg lengthening, Dog

## Abstract

Peroneal neuropathy is one of the complications of orthopaedic leg lengthening. Methods of treatment include slowing of distraction and decompression both of which may lead to additional complications. The purpose of this study was to analyse the changes in histologic peroneal nerve structure during experimental orthopaedic lengthening using various modes of manual or automatic distraction. The obtained data provide the basis for better understanding of peroneal neuropathy pathogenesis and refinement of prophylaxis and preventive treatment protocols. Four experimental models of canine leg lengthening using the Ilizarov fixator were studied: 1 (*n* = 10)—manual distraction—1 mm/day divided into four increments; 2 (*n* = 12)—automatic distraction—1 mm/day in 60 increments, 3 (*n* = 9) and 4 (*n* = 9)—increased rate of high frequency automatic distraction: 3 mm/day in 120 and 180 increments, respectively. In peroneal nerves semi-thin sections cross-sectional fascicular areas, content of adipocytes in epineurium, endoneurial vascularisation, morphometric parameters of nerve fibres were assessed by computerised analysis at the end of distraction and of consolidation periods and 30 days after fixator removal. In Groups 1–2 massive nerve fibre degeneration along with epineural vessels obliteration was revealed in two cases from 22, whereas in Groups 3–4 there were 10 from 18 (*p* < 0.01). Injuries of perineurium and endoneurial vessels were noted in Group 3, and long-lasting thinning of nerve fascicles in Group 4. The decrease in epineurial fat tissue was revealed in all groups, more drastic in 3. Modifications and injuries of nerve sheaths and blood vessels depending on distraction rate and frequency contribute to peroneal neuropathy. Its mechanical, circulatory and metabolic causes are discussed.

## Introduction

Orthopaedic limb lengthening is used to correct congenital and posttraumatic length discrepancies or to heighten stature [1–7]. Complications include disturbance to nerve functions [8–12]. Predominance of peroneal neuropathy is notable amongst all nerves clinically and in animal electro-physiologic research [12]. Anatomical predispositions of this condition were well studied [13–15]. Recovery of peroneal nerve dysfunctions in patients undergoing orthopaedic limb lengthening is slow or partial [11]. To prevent irreversible changes in neuromuscular system Nogueira and Paley [10] have developed two-staged protocol of slowing distraction and surgical decompression of the nerve. The first stage may lead to disturbances in bone regenerate formation. The second may add complications, specified by the authors [10] as including additional iatrogenic nerve injury, haematoma and infection. The aetiology of peroneal neuropathy associated with leg lengthening includes baseline nervous dysfunction in posttraumatic cases—where the trauma may have induced some damage [11], mechanical entrapment or overstretching and ischaemic condition during the operation or distraction period. Eighty-four per cent of the nerve lesions occur during distraction [9]. Subclinical peroneal nerve damage seen with electrophysiological testing is very frequent [8] and, even in the healthy adolescent, the peroneal nerve may be overcome with clinically evident neuropathic changes during a growth spurt [16]. During orthopaedic limb lengthening artificially induced tissue growth necessary for adaptation to the increased length depends on the rate and rhythm of distraction. The rate of 1 mm/day divided into 4 or 60 increments was optimal for bone regenerate formation [1] but such method of lengthening is very time-consuming. Distraction rates exceeding 2 mm/day may lead to the failure of callus formation [17] and other undesirable effects. In particular, a distraction rate 3 mm/3 times daily increases limb pain and tenderness as compared to distraction of 1 mm/1 time a day [18]. Ilizarov [1] concluded “that the greater the distraction frequency, the better the outcome”. Recently it was found that in conditions of automated fast high frequency distraction dividing a 3-mm round the clock rate in 120 increments the bone regenerate formation and consolidation were successfully accelerated compared to 1 mm daily rate [19]. Histological analysis of peroneal nerve changes associated with various modes of leg lengthening is absent, and this information is necessary for refinement of prophylaxis and preventative treatment protocols. We undertook to assess the severity and causes of peroneal neuropathy in four experimental models of canine leg lengthening.

## Materials and methods

Nerve samples were collected during experiments which were performed originally for the assessment of bone regenerate formation in different modes of experimental leg lengthening. Experiments were performed in consent with Principles of Laboratory Animal Care (NIH Publication no. 85-23, revised 1985) with approval from our institutional ethical committee. A total of 44 mongrel adult dogs that weighed 20–25 kg with 18–20 cm leg length were used in this study. Three animals formed the intact group, and all the rest were operated. Transverse shin bones osteoclasia in the middle of the diaphysis and osteosynthesis by an Ilizarov fixator were performed in all experimental groups. The lengthening protocol involved a 5-day latent period. In group 1 (*n* = 10) the lengthening was by manual movement of graded traction at the rate of 1 mm/d in 4 increments of 0.25 mm, performed for 28 days giving a total of 28 mm lengthening (15% increase in the initial length of shin bones). The fixator was removed after the bone healed (30 days of fixation). The animals were euthanised and the material for histology was obtained at the end of distraction (postoperative day 33), at the end of consolidation phase and 30 days after the fixator removal. In Group 2 (*n* = 12) the leg lengthening with carried out with an automated distractor. The distraction protocol involved 5-day latency as in Group 1, and 28 days of automatic distraction with speed of 1 mm/day in 60 increments; the animals were euthanised at three time-points as in Group 1. In Groups 3 and 4 the distraction rate was 3 mm/day in 120 and 180 increments (increment length 0.025 and 0.017 mm), respectively, with a total of 28 mm lengthening achieved in 10 days, and the fixator removed after bone consolidation (fixation 30 days only). The animals were euthanised at the end of distraction period (postoperative day 15) and 30 days after the fixator removal. The sciatic nerve in the thigh and its main branches in the leg was dissected in the operated and contralateral limbs. Parts of the peroneal nerve were excised, subjected to aldehyde-osmium fixation and embedded in Araldite. Semi-thin (0.5–1.0 µm) sections were prepared using “Nova” ultra-tome LKB (Sweden), stained with Toluidine Blue and Methylene Blue-Basic Fuchsin. Tissue sampling and processing, sectioning, staining and histomorphometric measurements were made according to standard methodology. In total, transverse slices of peroneal nerves, fascicular areas (*A*_f_) and occupational areas of epineural adipocytes were assessed. In 25 non-overlapping fields of the endoneurial compartment from each nerve, collected in a systematic random order, the numerical densities of endoneurial vessels (N_A_mv), myelinated and unmyelinated nerve fibres (N_A_mf and N_A_uf) were evaluated. About 400 samples of myelinated fibres for each nerve site were made and morphometric parameters—diameters of myelinated nerve fibres, their axons and myelin sheath thickness—were measured. The proportion (per cent) of degenerated myelinated nerve fibres in the samples was counted. Histomorphometric data were evaluated for statistical differences using the software package Attestat Program, version 9.3.1 (developed by I. P. Gaidyshev, Certificate of Rospatent official registration No. 2002611109) using the unpaired *t* test, Mann–Whitney *U* test and Barnard test, designed for small samples (*n*1, *n*2 ≥ 3). A significant value of *p* < 0.05 was set a priori.

## Results

Symptoms of neurological deficiency (plantar hyperflexion of tarsus, knuckling of digits, hypoalgesia) were evident during the second half of the distraction phase in five from 10 animals of Groups 1, in one animal of Group 2, and in five from nine animals in groups 3 and 4. These signs were intermittent and disappeared during the fixation period. Symptoms of neuropathic pain were absent. Some of the dogs in Groups 1–2 and the majority of animals in Groups 3–4 did not use the operated limb during distraction period. In the fixation phase the muscle tone of the operated limb improved progressively but the range of joint motions was decreased, especially in Groups 3 and 4. After fixator removal the ranges of joint motion were improved. As a whole, there were minimal functional disturbances in Group 2 and maximal in Group 3 as presented in Table 1. After the animals were euthanised the sciatic nerve in the thigh was exposed; its peroneal and tibial branches were followed to the distal level of the leg. Malposition of wires and the abrupt changes of nerves shape or diameter were not found but the fibrous arch surrounding the peroneal nerve in lengthened leg was thicker.

**Table 1:** Quantification of functional deficit

Parameter-time point/group	Number of dogs that used the operated limb/number of dogs in group				Range of motion (% from normal) at 30 days after the fixator removal	
					Knee joint extension (%)	Ankle joint extension
	½ D	D	F	WA		
1 (1 mm/day in 4 steps)	4/10	2/10	4/6	3/3	100	60%
2 (1 mm/day in 60 steps)	11/12^1,3,4^	10/12^1,3,4^	6/6	3/3	100	100%
3 (3 mm/day in 120 steps)	3/9	0/9	5/6	3/3	80	Stiffness
4 (3 mm/day in 180 steps)	0/9	0/9	6/6	3/3	90	30–60%

*½ D* the middle of distraction period, *D* the end of distraction, *F* fixation period, *WA* without apparatus

^1,3,4^—Differences of Group 2 from Group 1, 3, 4 (*p* < 0.05)

**Fig. 1:**
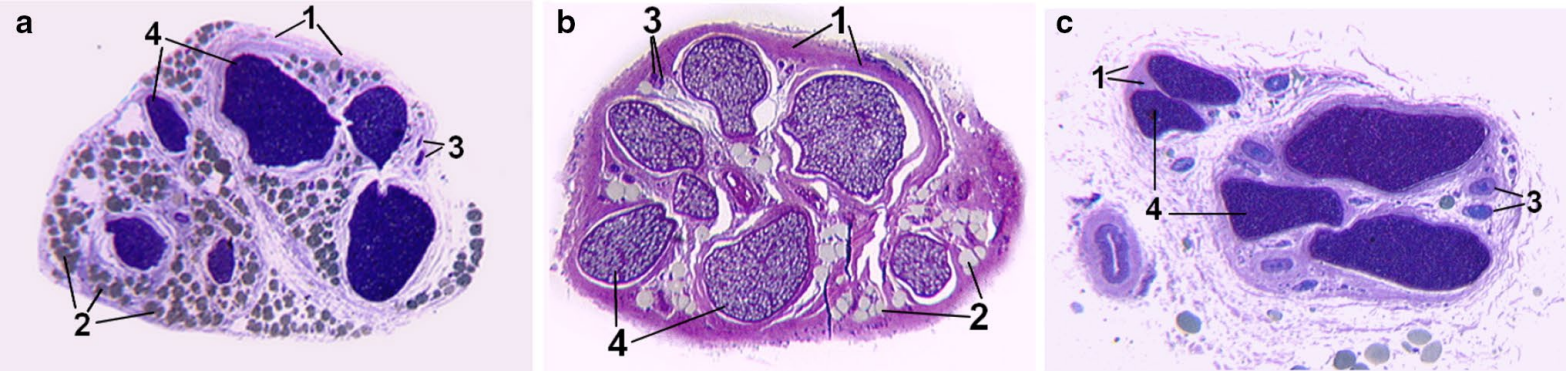
Total semi-thin sections of superficial peroneal nerve. **a** Total nerve section from intact dog, **b** total nerve section from canine lengthened leg, Group 2, 30 days after fixator removal, **c** total nerve section from canine lengthened leg, Group 1, 30 days after fixator removal. (1)—Collagen fibres in epineurium, (2)—fat cells (adipocytes) in epineurium, (3)—epineural vascular bundle (small artery and vein), (4)—nerve fascicles. Instrumental magnification ×31.25

**Fig. 2:**
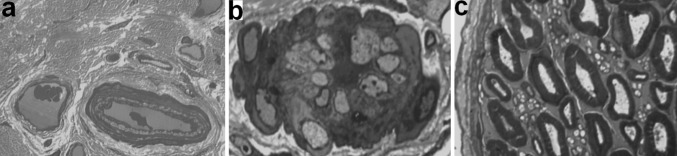
Fragments of lengthened nerve semi-thin sections from dogs of Group 1 (**a, b**) and 2 (**c**)**. a** Normal structure of small artery and vein in epineurium, **b** lumen obliteration in epineurial artery (at the end of distraction), **c** preserved structures of perineurium, endoneurium and nerve fibres (after fixator removal). Magnification ×1250

**Fig. 3:**
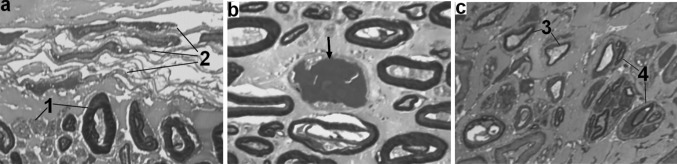
Fragments of lengthened nerve semi-thin sections from dogs of Group 3. **a** Degeneration of nerve fibres (1) and disintegration of perineural cells layers; **b** necrotic changes in endoneurial vessel (arrow) at the end of distraction; **c** regenerating nerve fibres (after fixator removal): some of them with signs of secondary degeneration (3), some are grouped in clusters (4)

**Fig. 4:**
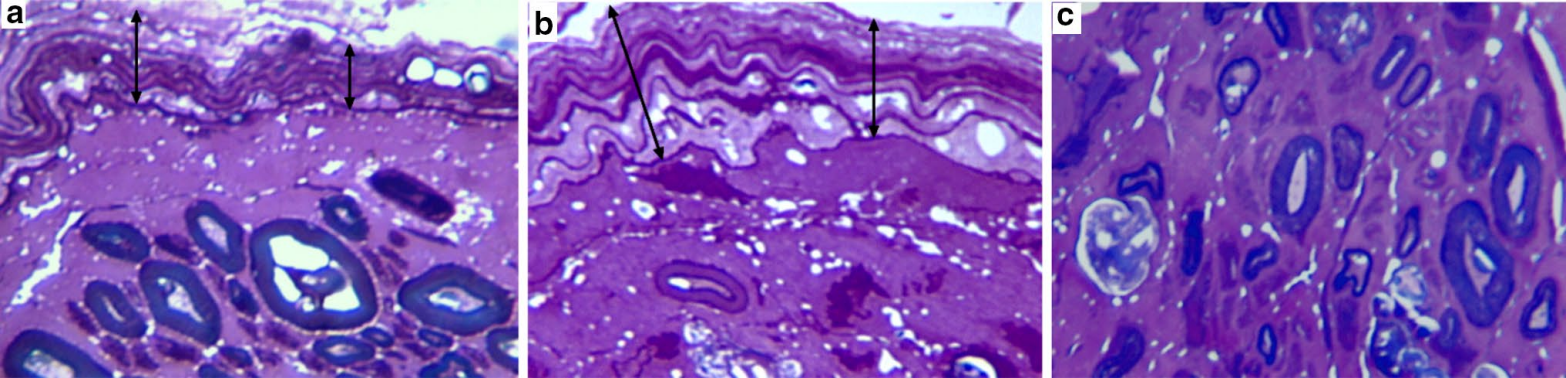
Fragments of lengthened nerve semi-thin sections from dogs of Group 4. **a, b** Perineurium (double-edged arrows) at the end of distraction (**a**) and fixation (**b**) periods; **c** endoneurium with few survived and lot of degenerated and regenerating nerve fibres

In comparison with intact nerves the epineurium in nerves from lengthened legs contained more collagen and less adipose tissue. Transverse sections areas of lengthened nerves did not differ significantly from contralateral ones. At the end of distraction the area occupied by epineural adipocytes decreased in all Groups—approximately 60% down (Table 2). Later these values increased 1.5 times only in Groups 1 and 2. In Group 2 loss of epineural adipocytes in the lengthened nerve was not so severe as in other Groups, with clinical signs (Fig. 1b) and measured values in the fixation period (Table 2) showing a substantial reversal initially but with a later decrease at the end of the experiment. Epineural blood vessels of lengthened and contralateral nerves increased in number and size in comparison with intact nerves—especially in Group 2 (Fig. 1). Some blood vessels of lengthened nerves were flattened and had collapsed or obliterated lumens (Fig. 2b). In Groups 3 and 4 the number of obliterated epineurial vessels was higher although the epineurium was also hypervascularised. The perineurium in Groups 1 and 2 preserved its continuity and thin lamellar structure (Fig. 2c). In Group 3 the points of disintegration of the perineurial inner layers were revealed (Fig. 3a). In Group 4 the perineurium was oedematous (Fig. 4a, b).

**Table 2:** Mean occupational area of epineural adipocytes (%)

Intact	The end of distraction period		30 days of fixation in apparatus		30 days after fixator removal	
	Lengthened	Contralateral	Lengthened	Contralateral	Lengthened	Contralateral
30.4	*Group 1*—*1* *mm/day in 4 steps*					
	4.2	12.0	9.2	18	5.5	19.8
	*Group 2*—*1* *mm/day in 60 steps*					
	9.5	12.4	16.1	18.1	6.7	15.8
	*Group 3*—*3* *mm/day in 120 steps*					
	5.3	12.0	3.6	13.3	4.0	11.1
	*Group 4*—*3* *mm/day in 180 steps*					
	8.1	13.5	5.8	10.3	6.0	13.0

**Fig. 5:**
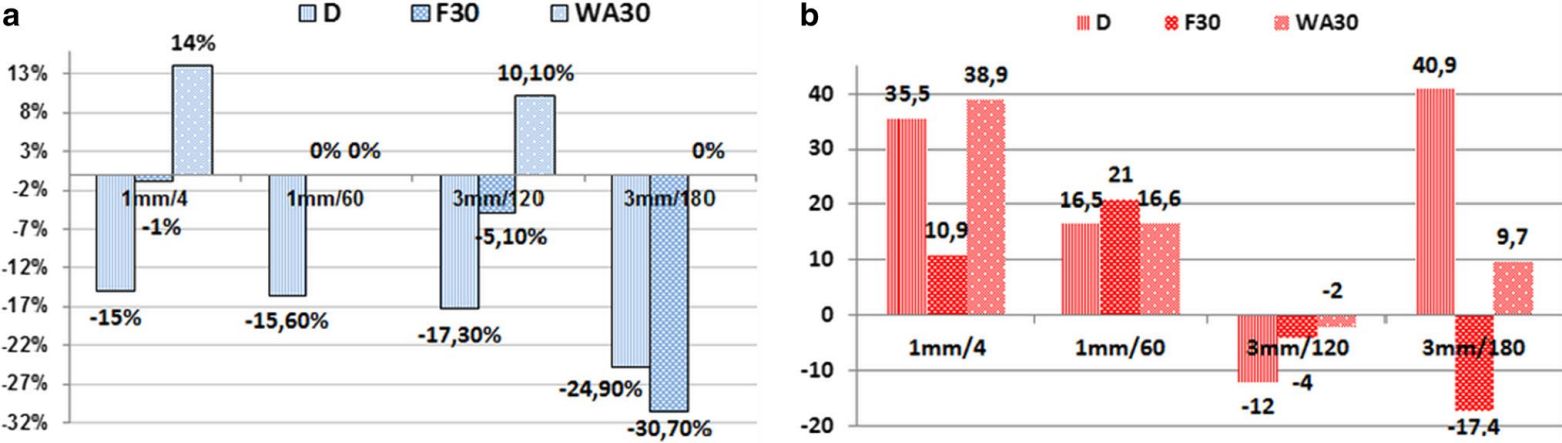
Fascicular areas and endoneurial vessels quantification at various time-points of experiment (D—the end of distraction; F—the end of consolidation phase; WA30—30 days after the fixator removal). **a** Per cents of differences of fascicular areas (*A*_f_) between nerves of lengthened (*l*) and contralateral (*c*) legs: (*A*_f_ *l*—*A*_f_ *c*)/*A*_f_ *c* × 100%. **b** Per cents of differences of endoneurial vessel numerical densities (N_A_mv) between nerves of experimental (*e*) and intact (*i*) animals: (N_A_mv-*e* − N_A_mv-*i*)/N_A_mv-*i* × 100%

The fascicular area of lengthened nerve at the end of distraction was smaller than contralateral one in all groups (Fig. 5a). The extent of fascicular thinning was approximately equal in Groups 1 and 2 but more prominent and longer lasting in Group 3 and especially in Group 4. At the end of experiment diameters of the lengthened nerves in Groups 1 and 3 were bigger than of contralateral ones. These findings were associated with points of endoneurial and sub-perineural oedema and endoneurium thickening. Numerical densities of endoneurial vessels in lengthened nerves (Fig. 5b) were increased in Group 1 and 2. In Group 3 the endoneurium was hypovascularised with signs of endoneurial microvessel necrosis (Fig. 3b). In Group 4 the diameter was increased at the end of distraction and after the fixator removal but at the end of fixation this was decreased significantly.

**Table 3:** Quantification of nerve fibres loss

Parameters-time point/group	Mean relative numerical density (N_A_-e* − N_A_-i**)/N_A_-*i* × 100%					
	Of myelinated nerve fibres			Of unmyelinated nerve fibres		
	D	F	WA	D	F	WA
1 (1 mm/day in 4 steps)	3.3	− 9.4	8.1	− 20.6	9.0	0
2 (1 mm/day in 60 steps)	7.2	4.6	− 6.4	2	7.9	3.13
3 (3 mm/day in 120 steps)	8.8	− 22.7	0	− 25.7	− 25.9	10.8
4 (3 mm/day in 180 steps)	− 24.8	− 31.0	4.9	− 1.5	− 4.3	1.5

N_A_-*e* values of lengthened nerve in experimental animals, N_A_-*i* values from intact animals

*D* the end of distraction, *F* fixation period, *WA* without apparatus

The proportion of degenerated myelin nerve fibres did not exceed 6% in Group 1 and 4% in Group 2. Relative numerical densities of myelin nerve fibres varied from − 9.4 to + 8.1% (Table 3). Only in two animals (single in every group) massive myelin nerve fibre degeneration (> 40%) occurred along with lumens obliteration in the largest epineural vessels. In Groups 3 and 4 the majority of animals (10 from 18, five in every group) overcame massive degeneration of myelin nerve fibres. Numerical densities of nerve fibres at the end of experiments were comparable with intact nerves due to remyelination and regeneration but most of regenerated nerve fibres possessed the signs of secondary axonal or Wallerian degeneration (Figs. 3c, 4c). In Groups 1 and 3 unmyelinated nerve fibres loss was more severe than in Groups 2 and 4. Morphometric analysis of myelinated nerve fibres populations revealed that in all animals the average axonal diameter was decreased at periods of distraction and fixation, but at the end of experiment in Groups 1 and 2 the differences to intact nerves were insignificant. Recovery of all morphometric parameters of myelin nerve fibres population was noted only in Group 2. In group 1 myelin sheath thickness and nerve fibre diameter remained significantly decreased. In Group 4 at the end of distraction and fixation periods diminution of axonal diameters and myelin sheath abnormalities (oedema of Schwann cells or hypo-myelination) were worse, but at the end of experiment the axonal diameters were better than in Group 3 and the average myelin sheath thickness was comparable with corresponding parameter of intact nerve (Table 4).

**Table 4:** Mean morphometric parameters (± standard error of mean) of myelinated nerve fibres populations

Parameters-time point/group	Myelinated nerve fibres diameters			Axonal diameters			Myelin sheath thickness		
	D	F	WA	D	F	WA	D	F	WA
0 (intact)	6.46 ± 0.07			4.39 ± 0.08			1.04 ± 0.04		
1 (1 mm/day in 4 steps)	5.37 ± 0.41^a^	6.09 ± 0.63^a^	5.90 ± 0.43^a^	3.69 ± 0.29^a^	4.44 ± 0.57	4.10 ± 0.10	0.84 ± 0.09^a^	0.98 ± 0.06	0.90 ± 0.17^a^
2 (1 mm/day in 60 steps)	5.56 ± 0.26^a^	5.62 ± 0.07^a^	6.17 ± 0.45	3.70 ± 0.53^a^	3.91 ± 0.09^a^	4.14 ± 0.16	0.92 ± 0.13^a^	0.85 ± 0.07^a^	1.01 ± 0.06
3 (3 mm/day in 120 steps)	5.95 ± 0.20^a^	4.45 ± 1.02^a^	4.64 ± 0.58^a^	3.56 ± 0.10^a^	2.82 ± 0.59^a^	2.98 ± 0.29^a^	1.20 ± 0.05^a^	0.81 ± 0.22^a^	0.84 ± 0.15^a^
4 (3 mm/day in 180 steps)	5.84 ± 0.29^a^	2.82 ± 0.04^a^	5.32 ± 0.82^a^	3.38 ± 0.08^a^	2.12 ± 0.04^ab^	3.32 ± 0.36^ab^	1.23 ± 0.10^a^	0.35 ± 0.00^ab^	1.00 ± 0.24^b^
0 (intact)	6.46 ± 0.07			4.39 ± 0.08			1.04 ± 0.04		

^a^Differences with intact nerve, ^b^Difference of Group 4 from Group 3 (*p* < 0.05)

*D* the end of distraction, *F* fixation period, *WA* without apparatus

## Discussion

Functional deficiency occurring during the leg lengthening has neuropathic, myopathic and arthropathic aspects. Even subclinical (asymptomatic) injuries of peroneal nerve may compromise functional rehabilitation. In this four-model experiment where distraction rates and frequencies differ, it has been shown that minimal functional deficit with full restoration of the operated limb strength and joint motion range was achieved in group with slow automated distraction (1 mm/60 times a day). Macroscopic signs of external local compression of the common peroneal nerve and its branches in anatomical sites known for impingement (e.g. fibular neck) were not found in any of these models. The same was noted by other authors in lengthening research carried out in the rabbit leg [18], but the possibility of wide-spread compression of nerves from thickened fascial structures cannot be excluded. To our knowledge, this is the first description of histological responses of peroneal nerve to limb lengthening. Experiments on instant stretching of the rabbit tibial nerve [20] revealed that “under tension the cross-sectional area of the fascicles is reduced, causing an increase in intra-fascicular pressure and consequent interference with intra-fascicular nutritive blood flow”; the “upper stretching limit” when all microcirculation in nerves ceases varied from 11 to 18%. In our research of gradual leg lengthening of 15% in the dog, some epineural veins and arteries with closed lumens were found along with an increase in the size and number of epineural vessels corresponding to an angiogenesis. In spite of this, in cases where there was obliteration of the largest epineural blood vessels, massive nerve fibre degeneration (indicating severe neuropathy) was revealed. This condition occurred rarely in groups with mild distraction and more often in groups with fast distraction (two cases from 22 against 10 from 18—*p* < 0.01). Kihara et al. [21] showed that if the peripheral nerve is rendered ischaemic by micro-embolisation, it can be rescued effectively from degeneration by hyperbaric oxygenation. We suggest that gradual lengthening ensures a wider temporal window for anti-hypoxic treatment and this technique may be successful in series with slower rates of distraction. In groups with fast distraction the nerve sheaths injuries were more complex. Besides obliteration of epineural vessels, perineurium and endoneurial vessels ruptures was found in Group 3 (3 mm/day in 120 steps) and permanent wrinkling of the perineurium in Group 4 (3 mm/day in 180 steps) in spite of the very small incremental length. The prognosis of this type of neuropathy is doubtful because of the secondary Wallerian and axonal degeneration. It is known that the epithelial membrane antigen positive perineural cells [22] constitute a metabolically active barrier [23] responsible for endoneurium homeostasis. In all experiments we revealed a decrease in epineural fat tissue both in lengthened and in contralateral nerves in comparison with intact nerves and this corresponded to weight loss in the operated animals. Paley [2] described a lowering of appetite, weight loss and depression in all patients with limb lengthening at period of distraction with quick recovery in consolidation period. In our animal research a partial recovery of adipocyte content in epineurium of the contralateral nerve was noted only in the mild distraction groups; these were associated with small areas of myelinated nerve fibre degeneration. According to the literature, in various situations where there is rapid weight loss—including World War II prisoners, dieting, bariatric surgery—the increase in peroneal neuropathy rate is well documented [24] although the exact mechanism of such relation is unknown. Gene expression analysis suggests that the epineurium may regulate energy metabolism in Schwann cells and axons via a supply of both fat metabolites and adipokines [25]. On other hand, loss of epineural and subcutaneous fat may lead to increased peroneal nerve susceptibility to compression. We found that in all cases adipocytes content in the epineurium of the lengthened nerve was smaller than in contralateral. The biggest diameters during the fixation period was noted in the group with best nerve fibres survival (Group 2—1 mm/day in 60 steps) but, even in this group at the end of experiment, loss of fat tissue in the epineurium of the lengthened nerve was dramatic and accompanied with 6% loss of myelinated nerve fibres. Consequently, structural variations in the histomorphology of the peroneal nerve during leg lengthening depend substantially on the distraction rate and frequency. The safety window for peroneal nerve survival that is exposed to a fast rate of distraction is not known but further research into the automated distraction of 3 mm daily carried out around the clock and divided into 180 increments appears better for the nerve than the usual rate of manual distraction 1 mm/4 times a day [26].

## Conclusion

The present findings suggest that the key prophylactic measures for peroneal neuropathy associated with leg lengthening include safe distraction regimen (1 mm/day in 60 steps), prevention of quick weight loss and possibly anti-hypoxia treatment.
